# Development of 3D Printable Cementitious Composites with the Incorporation of Polypropylene Fibers

**DOI:** 10.3390/ma14164474

**Published:** 2021-08-10

**Authors:** Jolien Van Der Putten, Attupurathu Vijayan Rahul, Geert De Schutter, Kim Van Tittelboom

**Affiliations:** Magnel-Vandepitte Laboratory, Department of Structural Engineering and Building Materials, Ghent University, 9052 Ghent, Belgium; Jolien.VanDerPutten@UGent.be (J.V.D.P.); Rahul.AttupurathuVijayan@UGent.be (A.V.R.); Geert.Deschutter@Ugent.be (G.D.S.)

**Keywords:** 3D concrete printing, fiber reinforcement, water film thickness, mechanical properties, durability

## Abstract

Similar to conventional cast concrete, printable materials require reinforcement to counteract their low tensile strength. However, as traditional reinforcement strategies are not commonly used in 3D print applications, fiber reinforcement can serve as an alternative. This study aims to assess the influence of different polypropylene fiber lengths (3 and 6 mm, denoted as M3 and M6, respectively) and dosages (0.1 and 0.3% volume fraction) on the workability, pore structure, mechanical and shrinkage behavior of 3D printable cementitious materials. Fresh state observations revealed that the addition of a higher fiber volume decreased the workability of the material, irrespective of the fiber length as a result of the lower water film thickness (WFT). In hardened state, a marginal increase in total porosity could be observed when adding fibers to the mix composition. In addition, the flexural strength was found to increase with the addition of fibers, while no significant difference was observed in compressive strength. The increase in flexural strength was more pronounced in the case of longer-sized M6 fibers. Finally, the total drying shrinkage behavior was evaluated using mold-cast prisms. The addition of M6 fibers showed no beneficial effect in reducing total free shrinkage, while a reduction in total free shrinkage was observed when using M3 fibers.

## 1. Introduction

Extrusion-based manufacturing techniques have become more and more integrated in the construction industry due to their many advantages: reduced construction time and material consumption, the increased geometrical freedom and complexity. The most common among the extrusion-based manufacturing techniques is 3D concrete printing (3DCP). It is a technique of manufacturing structural elements by the layer wise deposition of concrete using a concrete 3D printer [1,2].

Unlike conventional cast concrete, the mechanical behavior of 3D printed elements is anisotropic due to the presence of interfaces and its mechanical strength can be lower compared to mold cast specimens, depending on the loading direction [3,4]. Therefore, to increase its flexural capacity and also to have a more ductile post-cracking behavior, additives like fibers can be beneficial in 3D printed structural components [2]. Many printable concrete formulations incorporate plastic fibers such as polypropylene, polyvinyl alcohol [5] and even other fibers like basalt, carbon, and glass [5,6,7,8]. Printable strain-hardening composites have also been developed by a few researchers using high volume substitution (1–2%) of fibers such as polyvinyl alcohol and high-density polyethylene [9,10,11,12].

Another aspect related to the printable material is the high shrinkage cracking potential. This can be attributed to various reasons such as the lack of formwork, the high binder content, the use of additives like silica fume in the mixture composition, and the high surface area-to-volume ratio in the slender printed elements [2]. Shrinkage could be mitigated by a proper internal curing by superabsorbent polymers or by the application of shrinkage reducing admixtures. Another strategy to mitigate shrinkage is by providing fiber reinforcement in the cementitious material. The beneficial role of adding fibers can be attributed to several reasons: they reduce crack formation, they improve the strain capacity of the fresh mixture, they bridge the stress over the crack surfaces and mitigate micro crack propagation from developing into actual plastic shrinkage cracks [13,14,15,16,17].

Although the addition of fibers improves the properties in the hardened state (i.e., increased tensile strength, decreased cracking potential, etc.), their addition would also have certain adverse effects on the fresh properties. Due to their hindrance to deformation and movement of the matrix, the addition of fibers decreases the workability and increases the water and paste demand of the composition [18,19]. This could be detrimental with regards to the printability requirements as the material requires sufficient flowability during extrusion [2]. In addition, when the material is extruded, it has to retain its shape without relevant deformations.

Aiming to develop a printable material including polypropylene fibers, this study focuses on the influence of different polypropylene (PP) fibers with various lengths (i.e., 3 and 6 mm) on the fresh and hardened properties of 3D printable materials. In the fresh state, the addition of fibers on the workability is examined by using the flow table test. The water film thickness theory is then applied to further assess the rheological behavior. To assess the mechanical behavior, specimens were extracted from two-layered printed elements and subjected to compression and flexural tests. Finally, the influence of fiber addition on the porosity was evaluated by using mercury intrusion porosimetry (MIP) and shrinkage behavior was assessed by using prismatic specimens.

## 2. Methodology

### 2.1. Materials and Mix Compositions

**Figure 1:**
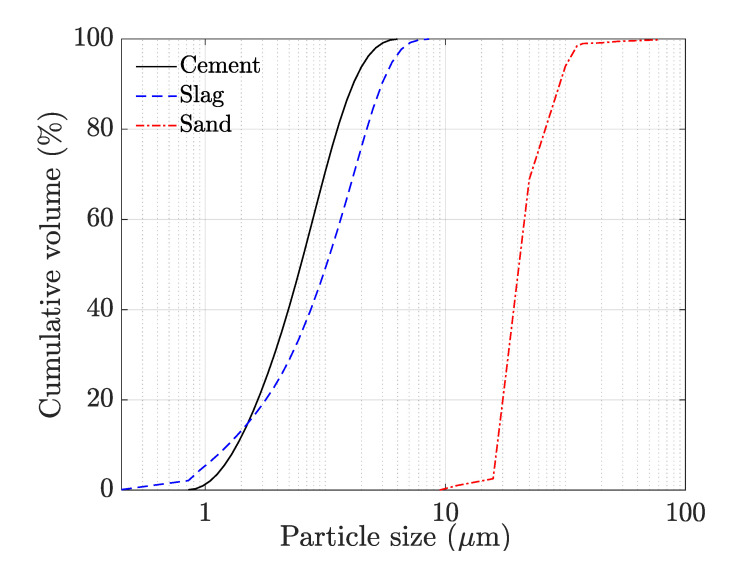
Particle size distribution of the binders and the aggregate.

CEM I 52.5 N Portland cement (conforming to NBN EN 197-1 [20]) was used in the current study. Ground granulated blast furnace slag (conforming to EN 15167-2 [21], supplied by ECOCEM (Moerdijk, The Netherlands) was used as a mineral admixture. The absolute density of the cement and slag equals 3.15 g/cm³ and 2.80 g/cm³, while the Blaine’s surface area of these materials is equal to 408 m²/kg and 428 m²/kg, respectively. Their chemical composition is shown in Table 1. Sand particles with a maximum size of 2 mm and an absolute density of 2.65 g/cm³ were used in the study. The particle size distribution of all the granular materials is visualized in Figure 1. This figure represents in addition also the similar particle size of the slag and cement used within this study. Chemical admixtures used include a polycarboxylic ether-based superplasticizer and a methyl cellulose-based viscosity modifying agent (VMA). The superplasticizer was used in a liquid form (34% solid content), while VMA was used as a solid. Two types of polypropylene fibers with varying length (designated as M3 and M6, based on their dimensions) were used in the study. Their properties are summarized in Table 2.

The design of the control mixture was based on previous work [22]. The control mixture had an aggregate-to-powder ratio of 1.2 and a water-to-powder ratio of 0.35. Both parameters can affect the paste volume fraction, which is considered as a critical parameter that affects both the fresh and hardened properties of concrete. The latter phenomena are for example revealed by Chu [7], who showed that the cementitious paste volume can affect the wet density, the mortar film thickness as well as the mechanical properties such as compressive, flexural, and tensile strength. 

The VMA dosage equaled 0.1% of the powder content for all mixtures. To evaluate the influence of the fiber addition, 0.1% and 0.3% volume percentage of both M3 and M6 fibers were added to the control mixture. The superplasticizer dosage for the control mixture was 0.35% of the powder content, however, higher superplasticizer dosages were required for fiber containing mixtures to ensure the same workability as the control mixture. The details of the different mixtures are summarized in Table 3.

All mixtures were prepared using a planetary Hobart mixer of 1.5-L capacity. The dry material was first mixed for 1 min. Then, the water, being mixed beforehand with the superplasticizer, was added. After 2 min of further mixing, the VMA was added and the mixing was continued for 2 more minutes. For the mixtures containing fibers, the mixing was stopped after 1 min of VMA addition. The fibers were scattered on top, and then mixing was resumed for the remaining duration of 1 min.

### 2.2. Assessment of Workability

The flow table test, in accordance with ASTM C1437-20 [23], was used to assess the workability. Immediately after mixing, the fresh concrete was poured in the mold in 2 layers, with each layer compacted 20 times using a tamping rod. The mold was then lifted, and the flow table was raised and dropped 20 times. The spread diameter of the printable material was measured in six directions. For each mixture, the experiment was conducted twice, and the average value is reported.

### 2.3. Printing Trials

**Figure 2:**
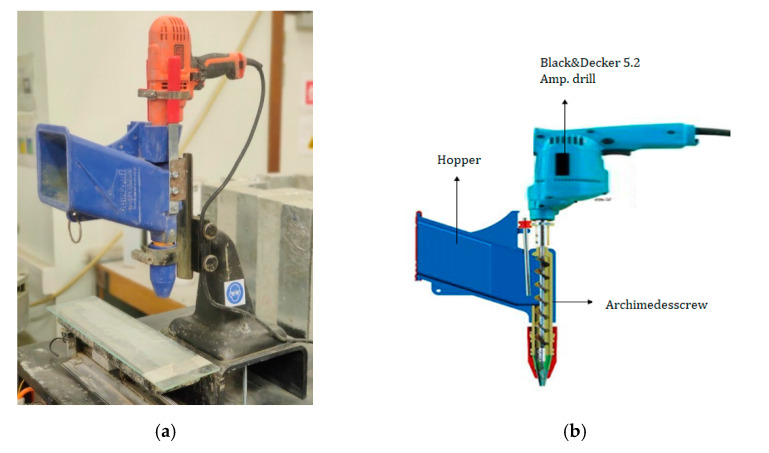
(**a**) Print device used in the current study, (**b**) equipped with a Quickpoint mortar gun with Archimedesscrew.

After mixing, the cementitious material was inserted into an in-house developed print equipment (Figure 2a)used to simulate an extrusion-based 3D printing process and composed of a Quickpoint mortar gun (Figure 2b), a Black and Decker 5.2 Amp drill build-in and an auger to move the cementitious material towards the cylindrical nozzle (18 × 28 mm²). The mortar gun was mounted vertically on a movable building platform and could be altered in height. The rotational speed of the auger was chosen in accordance with the applied printing speed, the translational motion of the building platform was controlled by a complementary software program. Within the scope of this research, the printing speed equals 1.7 cm/s. The layer height and length were fixed at 10 mm and 320 mm, respectively.

Sample preparation started by the extrusion of a substrate layer (l = 320 mm) After changing the vertical position (Z-position), the second layer was extruded on top of the previous one. The latter was deposited at the same horizontal position to ensure a similar time gap in between every position of the printed layer.

### 2.4. Determination of Compressive Strength

The procedure used for the compressive strength measurements was based on previous work by Van Der Putten et al. [24]. After a curing period of 28 days in standardized conditions (20 ± 2 °C temperature and 60 ± 5% relative humidity), small cylindrical samples (Ø = 14 mm, h = 20 mm) were drilled from the original two-layered printed elements. The top and bottom surface were smoothened before testing to ensure parallel loading surfaces. Additionally, 3 mm thick high-density fiberboard plates were put in between the loading frame and the specimen. Compressive strength measurements were performed perpendicular to the print direction under load control (Walter + Bai DB 250/15) with a loading rate of 100 N/s. The compressive strength was determined as follows:

(1) $$\mathrm{Compressive}\;\mathrm{strength}\;\left(N/\mathrm{mm}²\right)=\frac{4W}{{\pi d}^{2}}$$

where W (N) denotes the failure load and d (mm) denotes the diameter of the cylinder measured using a vernier caliper.

### 2.5. Determination of Flexural Strength

**Figure 3:**
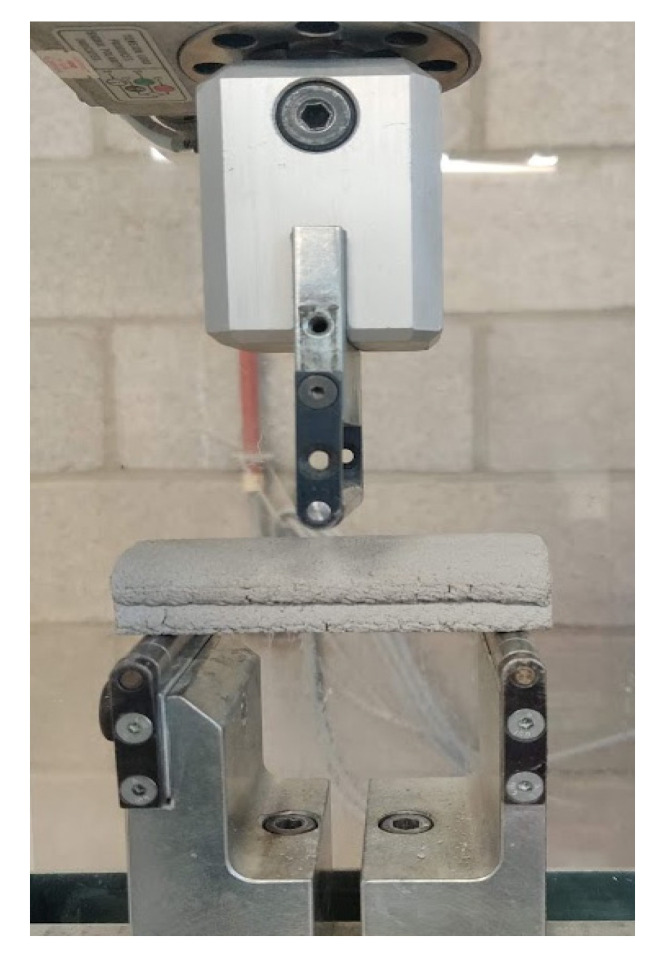
Three-point bending test set-up.

The 28-day flexural strength was measured using 100 × 20 × 20 mm^3^ beams extracted from the printed two-layer elements (Figure 3) and assessed using a three-point bending test with a free span of 80 mm. To obtain the beam specimens, 320 mm long two-layer specimens were first printed. Then, 100 mm beams were cut-out in the fresh state using a chisel. Both the printing and the curing of the specimens were done in an environmental control room of 20 ± 2 °C temperature and 60 ± 5% relative humidity. The loading rate for the flexural test was in accordance with NEN EN 196-1 [20,21]. The flexural strength was obtained as follows:

(2) $$\mathrm{Flexural}\;\mathrm{stregnth}\;\left(N/\mathrm{mm}²\right)=\frac{3\mathrm{Wl}}{2{\mathrm{bd}}^{2}}$$

where l, b, and d denote the length, width and depth of each beam (mm), measured accurately using a vernier calliper, and W (N) represents the failure load. For each case, a total of three beams were tested, and the average value is reported.

Please note that in the current study, the flexural tests are performed in a load-controlled manner. On the contrary, the flexural tests in a displacement control manner could also assess the post cracking characteristics based on the load-displacement curves. However, it must be noted that the differences in the load-displacement can also arise from the slight differences in the dimensions of the printed beam samples. Due to this limitation, analysis using the load-displacement curves could not be attempted. Only the peak stresses are being compared for the different mixtures. For this, the flexural test in a load-controlled manner was deemed adequate.

### 2.6. Pore Structure Assessment by Mercury Intrusion Porosimetry

The porosity of the printed specimens at the bulk, was investigated based on mercury intrusion porosimetry (MIP) measurements (Pascal 140 and 440 series, Thermo Fisher scientific Inc., Eindhoven, The Netherlands). After curing until 28 days in standardized conditions (20 ± 2 °C, 60 ± 5% RH), small cylindrical samples (Ø = 14 mm, h = 20 mm), drilled from an original two-layered printed element, were cut into smaller pieces taken from the bulk concrete (±15 mm³). These specimens were immersed in 2-propanol for 48 h to stop hydration. During the measurements, the maximum pressure was limited until 200 MPa to avoid crack formation. As a specific pressure corresponds to an aperture of a pore, and the amount of mercury intrusion approximates the pore volume, the number of pores and pore size could be determined.

### 2.7. Shrinkage Measurements

Shrinkage measurements on printed specimens are not standardized yet and are therefore performed using mold cast specimens, according to NBN B15-216 [25]. For each mixture, a series of 3 prismatic mold-cast elements (160 × 40 × 40 mm³) were fabricated per mix composition. All specimens were demolded after a hardening period of 24 h. Both casting and storage of the specimen was in a controlled environmental condition (20 ± 2 °C temperature and 60 ± 5% relative humidity). Two metallic measuring points were fixed at a gauge length of 100 mm on two of the opposite sides of each prism specimens. The length change in the original gauge length was obtained using a DEMEC meter after 1, 3, 7, and 28 days.

## 3. Results and Discussions

### 3.1. Assessment of Workability

To assess the influence of fiber addition on the workability, different dosages of M3 and M6 fibers were added to the control mixture. The flow spread diameters, obtained for the different compositions, are summarized in Table 4. With an increasing dosage of both fiber types, the flow spread diameter decreases.

Other researchers have previously reported a similar reduction in flow value with the addition of polypropylene fibers for conventional concrete [26]. To further assess the influence on workability, the thickness of the water film that coats on all the solids in the mixture, including the fibers, called the water film thickness (WFT), can be computed [26,27,28]. The WFT is given by:

(3) $$\mathrm{WFT}=\frac{\mu_{w}^{\prime }}{A_{s}}$$

where $\mu_{w}^{\prime }$ is the excess water ratio and $A_{s}$ (m²/m³) denotes the total specific surface area of granular materials used in the mixture. $A_{s}$ is determined by taking the sum of the volume fraction of each solid, including the sand and the fibers, per unit volume of mortar multiplied with their corresponding specific surface area. The excess water ratio can be determined as follows:

(4) $$u_{w}^{\prime }=u_{w}-u_{\min}$$

In the above equation $u_{w}$ (−) is the volume ratio of water to the total solids in the mixture and $u_{\min}\;$(−) is the minimum void ratio given by:

(5) $$u_{\min}=\frac{1-\tau }{\tau }$$

where τ (−) denotes the packing density and is calculated by using the wet packing density approach given by Li et al. [29].

The WFT computed for the different mixtures is indicated in Table 5. The addition of the fibers resulted in no change or a marginal increase in the wet packing density of the mortar mixture. According to Equations (4) and (5), the increase in the wet packing density can cause an increase in the excess water ratio, which may contribute to the rise in the WFT. However, the increase in the volume fraction of the fibers causes an additional surface area per unit volume of the mortar mixture. This additional surface area increases the value of $A_{s}\;$(column 5 of Table 5), which, in turn, results in an overall decrease in the WFT (Equation (3)). These results are in agreement with previous studies performed on conventional concrete [26,27,28]. For instance, Li et al. [26] determined the slump value and spread flow diameter for cement mortars containing different volume fractions of polypropylene fibers. They observed that both the slump and flow value were found to be a decreasing exponential function of the WFT for a fiber of a given length and diameter.

Since the mixtures with 0.1 and 0.3% of M3 and M6 fibers showed a lower flow value (Table 4 these mixtures were not extrudable using the 2D printer used in the current study. Therefore, the superplasticizer dosage was increased for these mixtures to have the same flow value as the control mixture (approximately 16–17 cm). The adjusted superplasticizer content for these modified mixtures is indicated in Table 3. The modified mixtures are denoted as 0.1% M3, 0.3% M3, 0.1% M6, and 0.3% M6 and were subjected to further studies.

### 3.2. Porosity

**Figure 4:**
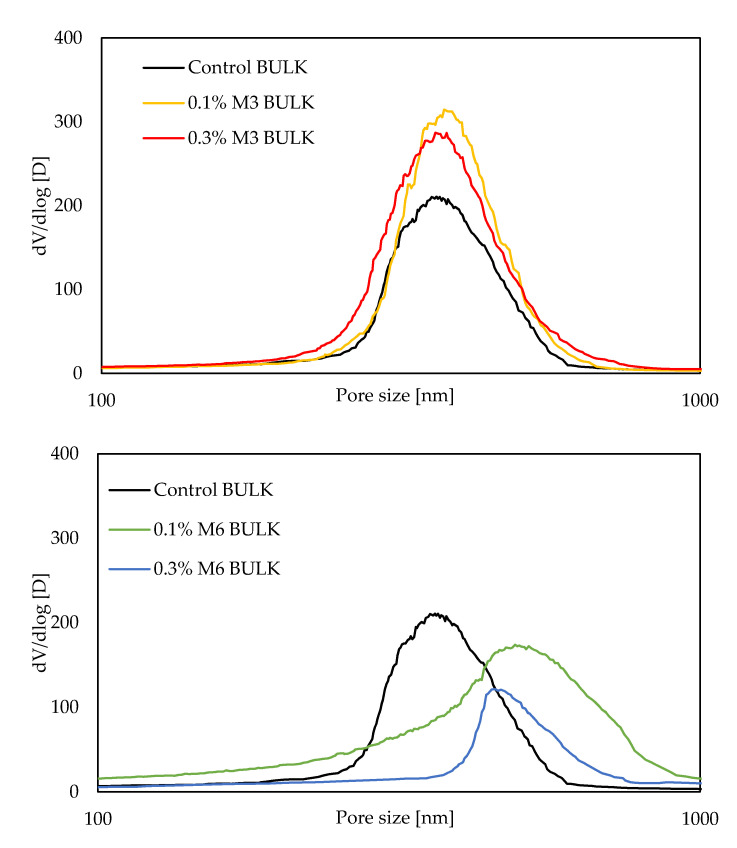
Pore size distributions of printed specimens with various mix compositions, measured at the bulk of the 3D printed concrete (*n* = 1).

Pore size distribution curves, obtained by MIP measurements at 28 days, are visualized in Figure 4. All mixtures, irrespective of the fiber type and dosage, followed a distinct unimodal pore size distribution. The different parameters derived from the pore size distribution curves are summarized in Table 6. Firstly, based on Table 6, it can be seen that the total porosity, in general, increases with the addition of both M3 and M6 fibers, irrespective of the added amount. A similar increase in porosity of printable cement-based materials has also been previously reported by other researchers [5,30]. However, total porosity solely is inadequate to form a strength-pore structure relationship [31]. Das and Kondraivendhan [32] indicated an empirical relationship where the compressive strength is proportional to (1-P), where P denotes the total pore volume fraction, and inversely proportional to the square root of the mean distribution radius (pore size measured at 50% volume intrusion of mercury). The effect of the fiber addition on the mean distribution is indicated in Table 6. Although the mean distribution radius does not change significantly with the addition of M3 fibers, an increase can be observed when the larger M6 fibers are used. The higher total porosity and the mean distribution radius for the M6 fibers may indicate a lower compressive strength for mixtures with 6 mm long fibers, compared to the other mixtures. Finally, the critical pore size, an important parameter related to transport properties [33,34], corresponds to the peak value of the differential pore volume curve (Figure 4). As shown in Table 6, the critical pore size follows a similar trend to that of the mean distribution radius, i.e., a higher value is observed for the larger M6 fibers compared to the other mixtures. This may indicate possible higher transport properties for the mixtures containing M6 fibers [33,34].

### 3.3. Compressive and Flexural Strength

**Figure 5:**
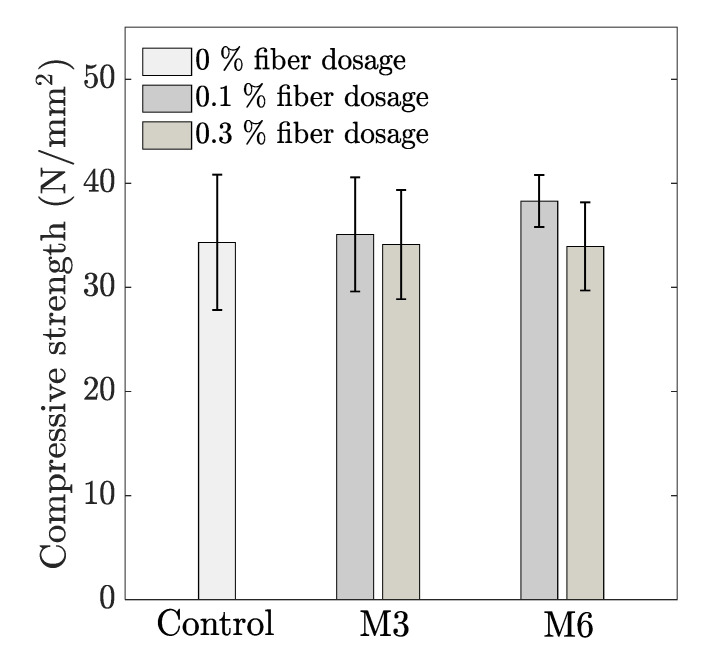
Influence of fiber addition on average 28-day compressive strength (error bar represents the standard deviation, *n* = 6).

The influence of the fiber addition on the compressive strength is represented in Figure 5. Although not significantly different, the addition of 0.1 vol% fibers slightly enhance the compressive strength, with the highest improvement in case of M6 (11% higher strength compared with the control mix). The longer PP fibers tend to align better parallel to the extrusion direction, which helps in crack bridging upon the action of the compressive force. Regardless of the fiber length, a further increase in fiber content resulted in a reduction of the compressive strength, which could be attributed to the slightly higher porosity as previously discussed in Section 3.2. Similar observations are also reported in the literature [5].

**Figure 6:**
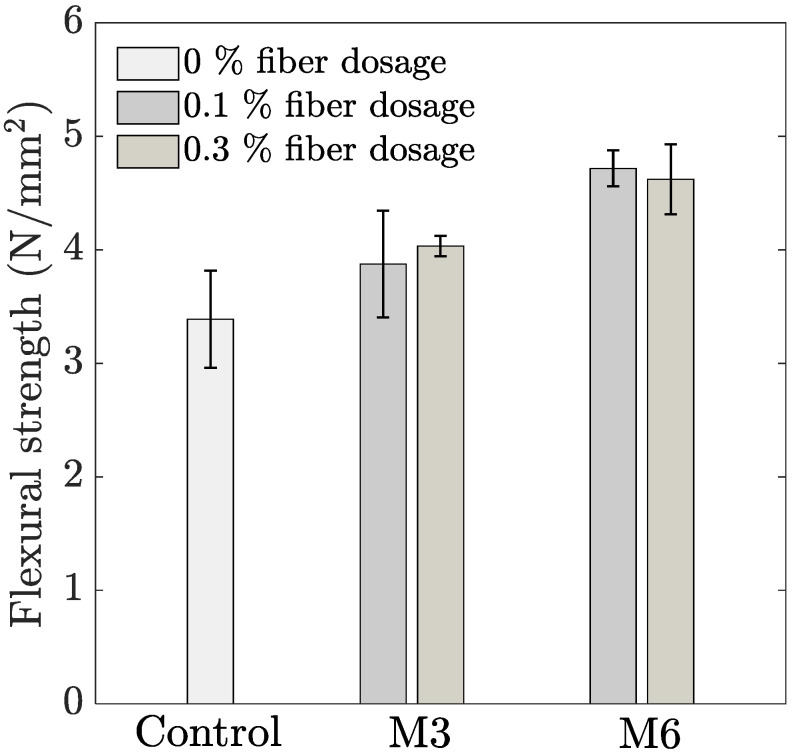
Influence of fiber addition on average 28-day flexural strength (error bar represents the standard deviation, *n* = 3).

The variation in flexural strength with the addition of both fiber types is represented in Figure 6. With an increasing dosage of M3 fibers, a gradual increase (14 and 19% for 0.1 and 0.3% fiber dosage, respectively) can be seen in the flexural strength compared to the control mixture. On the other hand, the flexural strength for M6 fibers increases by 39% for 0.1% fiber dosage, and thereafter, no further change is observed as the dosage is increased to 0.3% volume fraction. Similar improvement in flexural strength with the addition of PP fibers in printable concrete has also been reported by other researchers [2,5,30]. The closely spaced fibers obstruct micro crack propagation, delaying the onset of cracking and increasing the flexural strength [35,36]. Another aspect to be noted is that the addition of longer fibers creates a higher improvement in flexural strength compared to the addition of M3 fibers. This can be understood based on the analytical relationship given by Li [37], which suggests that the peak stress in flexural testing of concrete containing straight fibers is directly proportional to the volume fraction and the aspect ratio (length-to-diameter ratio) of the applied fibers. The higher aspect ratio of the M6 fibers compared to M3 fibers (i.e., 272 compared to 136, respectively) explains the higher flexural strength compared to shorter fibers when used at the same dosage.

### 3.4. Shrinkage Behaviour

**Figure 7:**
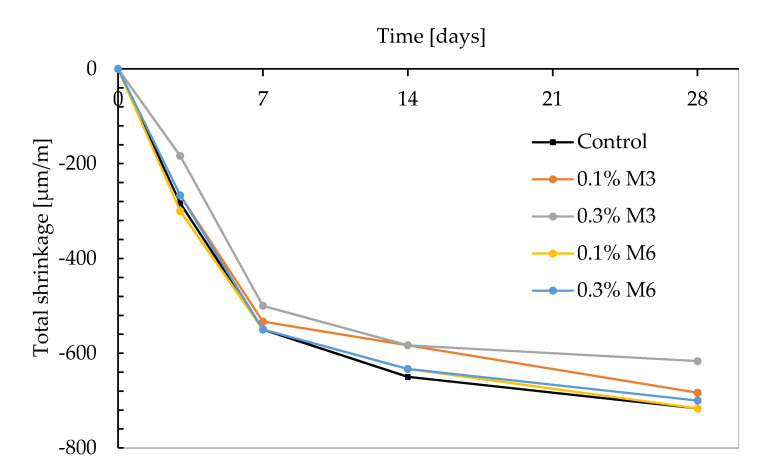
Total shrinkage of mixtures with and without the addition of fibers (*n* = 3).

**Figure 8:**
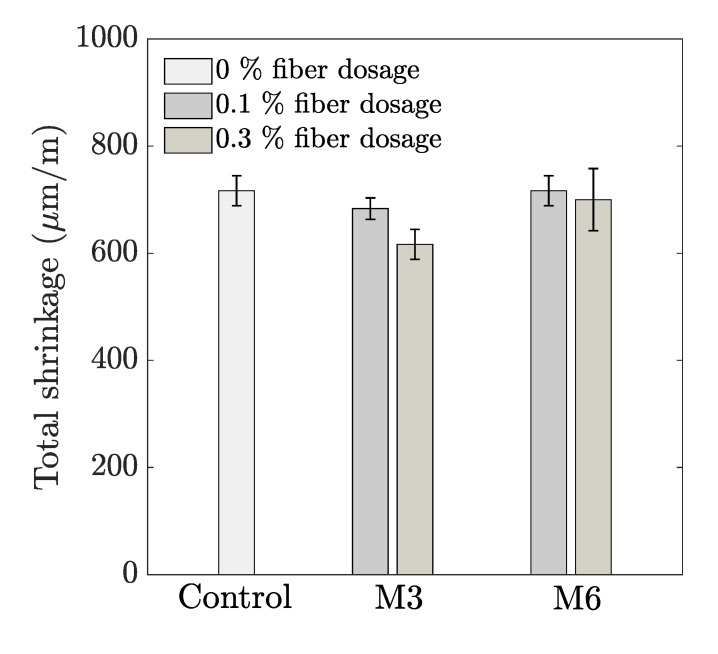
28-day cumulative total free shrinkage (*n* = 3).

Figure 7 represents the free shrinkage of the different mix compositions. For all test series, the shrinkage proceeds much faster in the first 7 days. The total cumulative strain at the end of the measuring period (i.e., after 28 days) is shown in Figure 8. It can be observed that the shrinkage behavior is comparable, irrespective of the fiber geometry, with 0.3% M3 as the only exception. For the latter test series, a reduction of approximately 100 µm/m could be observed compared to the control mix. For the smaller sized fibers, the reduction in total shrinkage is proportional to the fiber addition. However, the latter observation could not be distinguished in case of M6 fibers. The results of the total shrinkage can be related to the mechanical properties discussed before. The higher flexural strength in case of M3 and M6 mix compositions causes marginally higher resistances to tensile stress cracking and reduces the cracking potential. According to literature, smaller sized fibers are more effective in terms of shrinkage reduction. 

Fibers, in general, restrain the shrinkage even in the early stage of the hardening process of the cement matrix. This can be explained based on the bond between the fibers and the cementitious materials. As the bond strength between the fibers and the cement paste increases, a simultaneous increase in the overall stiffness of the fiber–cement paste composite will occur [38]. This creates a restraining effect which results in a reduction of the overall shrinkage. In the current study, it is seen that the smaller sized fibers tend to restrain the matrix to a higher extend and therefore, seem to be more beneficial with regard to shrinkage reduction. 

However, it must be noted that apart from the total free shrinkage strain, various other parameters such as the elastic modulus, the stress relaxation coefficient at the interface where the shrinkage deformation is being restrained, and the tensile strength can strongly influence the shrinkage cracking potential [38]. Although the free shrinkage deformation is lower for the M3 fiber compared to M6 (Figure 7), the flexural strength is found to be higher in the case of the longer M6 fibers (Figure 6). This can have a positive effect in increasing the resistance to shrinkage cracking potential. Further studies on shrinkage behavior under restrained conditions are required to gain further understanding in this regard.

## 4. Conclusions

The current study examines the feasibility of using polypropylene (PP) fibers to develop 3D printable cementitious composites. The general conclusions from the current study can be listed as follows:

An increased PP fiber volume fraction causes a decrease in the flow value, which can be explained based on the WFT theory. The addition of microfibers enlargers the surface area, thereby increasing total specific surface area per unit volume of solids. This increases the specific surface area, decreasing both the WFT and flow value of the concrete mixture.

The assessment of pore structure using MIP revealed that the total porosity increases, irrespective of the added fiber type. The addition of longer M6 fibers also increases, in addition, the mean distribution radius and the critical pore size, which may adversely influence the transport properties.

Considering the loading direction and fiber dosages in this study, no significant influence on the compressive strength could be observed for both fiber types. The flexural strength on the other hand showed an increase with an increased fiber dosage. For the longer M6 fibers, the increase was more significant. Fibers in cementitious matrices restrain the microcrack propagation during loading, and consequently cause a delay in the onset of flexural cracking and an increase in flexural strength.

The total free shrinkage decreases with the addition of shorter M3 fibers, while no significant reduction occurs when M6 fibers are used. The shrinkage reduction with the addition of fibers can be attributed to the deformation restrain caused by the introduction of fibers in the cement matrix.

## Figures and Tables

**Table 1:** Chemical composition of the cement and slag used in the study.

Oxide	Quantity (mass%)	
	Cement	Slag
CaO	64.30	37.97
SiO_2_	18.30	35.60
Fe_2_O_3_	4.00	0.37
Al_2_O_3_	5.20	13.12
MgO	1.40	7.24
SO_3_	3.50	7.24
(Na_2_O)_e_	0.32	0.74
LOI	1.40	0.95

**Table 2:** Properties of the polypropylene fibers used in the study.

Name	M3	M6
Length (mm)	3	6
Cross-sectional diameter (µm)	22	22
Specific gravity (g/cm³)	0.95	0.95

**Table 3:** Proportions of the mixtures.

Material	Quantity (g)				
	Control	0.1% M3	0.3% M3	0.1% M6	0.3% M6
Cement	450	450	450	450	450
Slag	450	450	450	450	450
Sand	1080	1080	1080	1080	1080
Water	315	315	315	315	315
Superplasticizer	2.81	3.15	3.33	3.42	3;78
VMA	0.91	0.91	0.91	0.91	0.91
Fibers	-	0.97	2.91	0.97	2.91

**Table 4:** Flow spread diameter of different mixtures (*n* = 3).

Mixture	Fiber Dosage (vol%)	Flow Spread Diameter (mm)
Control	-	173
M3	0.1	164
	0.3	159
M6	0.1	162
	0.3	153

**Table 5:** Effect of fiber addition on WFT.

Mixture	Fiber Dosage (%)	Wet PD τ (−)	Excess Water Ratio $u_{w}^{'}\;(-)$	Total Surface Area $A_{s}\;(m^{2}/m^{3})$	WFT (nm)
Control	0	0.70969	0.03389	1,103,784	30.7
M3	0.1	0.70276	0.01244	1,277,281	9.7
	0.3	0.69805	0.00283	1,623,816	1.7
M6	0.1	0.70453	0.01601	1,189,868	13.5
	0.3	0.69846	0.00367	1,361,807	2.7

**Table 6:** Pore characteristics obtained from mercury intrusion porosimetry for the bulk material (*n* = 1).

Specimen	Porosity (%)	Mean Distribution Radius (nm)	Critical Pore Size (nm)
Control	14.03	362.84	362.84
0.1% M3	16.40	373.54	314.72
0.3% M3	17.29	363.74	361.16
0.1% M6	18.59	484.86	492.67
0.3% M6	11.12	457.27	467.05

## Data Availability

The data presented in this study are available on reasonable request from the corresponding author.

## References

[1] Lim S., Buswell R., Le T., Austin S., Gibb A., Thorpe T. (2012). Developments in construction-scale additive manufacturing processes. Autom. Constr..

[2] Mohan M.K., Rahul A., De Schutter G., Van Tittelboom K. (2021). Extrusion-based concrete 3D printing from a material perspective: A state-of-the-art review. Cem. Concr. Compos..

[3] Le T., Austin S., Lim S., Buswell R., Law R., Gibb A., Thorpe T. (2012). Hardened properties of high-performance printing concrete. Cem. Concr. Res..

[4] Rahul A.V., Santhanam M., Meena H., Ghani Z. (2019). Mechanical characterization of 3D printable concrete. Constr. Build. Mater..

[5] Hambach M., Volkmer D. (2017). Properties of 3D-printed fiber-reinforced Portland cement paste. Cem. Concr. Compos..

[6] Ma G., Li Z., Wang L., Wang F., Sanjayan J. (2019). Mechanical anisotropy of aligned fiber reinforced composite for extrusion-based 3D printing. Constr. Build. Mater..

[7] Chu S., Li L., Kwan A. (2021). Development of extrudable high strength fiber reinforced concrete incorporating nano calcium carbonate. Addit. Manuf..

[8] Li L., Xiao B., Fang Z., Xiong Z., Chu S., Kwan A. (2021). Feasibility of glass/basalt fiber reinforced seawater coral sand mortar for 3D printing. Addit. Manuf..

[9] Figueiredo S.C., Rodriguez C.R., Ahmed Z.Y., Bos D., Xu Y., Salet T.M., Copuroglu O., Schlangen E., Bos F.P. (2019). An approach to develop printable strain hardening cementitious composites. Mater. Des..

[10] Ogura H., Nerella V.N., Mechtcherine V. (2018). Developing and Testing of Strain-Hardening Cement-Based Composites (SHCC) in the Context of 3D-Printing. Materials.

[11] Soltan D.G., Li V.C. (2018). A self-reinforced cementitious composite for building-scale 3D printing. Cem. Concr. Compos..

[12] Zhu B., Pan J., Nematollahi B., Zhou Z., Zhang Y., Sanjayan J. (2019). Development of 3D printable engineered cementitious composites with ultra-high tensile ductility for digital construction. Mater. Des..

[13] Swamy R., Stavrides H. (1979). Influence of fiber reinforcement on restrained shrinkage and cracking. J. Proc..

[14] Paillere A.M., Buil M., Serrano J. (1989). Effect of fiber addition on the autogenous shrinkage of silica fume. Mater. J..

[15] Soroushian P., Mirza F., Alhozajiny A. (1993). Plastic shrinkage cracking of polypropylene fiber reinforced concrete. Mater. J..

[16] Sargaphuti M., Shah S.P., Vinson K. (1993). Shrinkage cracking and durability characteristics of cellulose fiber reinforced concrete. Mater. J..

[17] Banthia N., Gupta R. (2006). Influence of polypropylene fiber geometry on plastic shrinkage cracking in concrete. Cem. Concr. Res..

[18] Martinie L., Rossi P., Roussel N. (2010). Rheology of fiber reinforced cementitious materials: Classification and prediction. Cem. Concr. Res..

[19] Sultangaliyeva F., Carré H., La Borderie C., Zuo W., Keita E., Roussel N. (2020). Influence of flexible fibers on the yield stress of fresh cement pastes and mortars. Cem. Concr. Res..

[20] NBN (2000). Cement-Part 1: Composition, Specifications and Conformity Criteria for Common Cements.

[21] British Standards Institution (2000). BS EN 15167-2:2018: Cement-Part 1: Ground Granulated Blast Furnace Slag for Use in Concrete, Mortar and Grout—Part 2: Conformity Evaluation, 2018.

[22] Mohan M.K., Rahul A., Van Tittelboom K., De Schutter G. (2021). Rheological and pumping behaviour of 3D printable cementitious materials with varying aggregate content. Cem. Concr. Res..

[23] ASTM (2020). Standard Test Method for Flow of Hydraulic Cement Mortar.

[24] Van Der Putten J., Deprez M., Cnudde V., De Schutter G., Van Tittelboom K. (2019). Microstructural Characterization of 3D Printed Cementitious Materials. Materials.

[25] NBN (1974). Proeven op Beton: Krimpen en Zwellen.

[26] Li L., Chu S., Zeng K., Zhu J., Kwan A. (2018). Roles of water film thickness and fibre factor in workability of polypropylene fibre reinforced mortar. Cem. Concr. Compos..

[27] Chu S., Ye H., Huang L., Li L. (2021). Carbon fiber reinforced geopolymer (FRG) mix design based on liquid film thickness. Constr. Build. Mater..

[28] Li L., Zeng K., Ouyang Y., Kwan A. (2019). Basalt fibre-reinforced mortar: Rheology modelling based on water film thickness and fibre content. Constr. Build. Mater..

[29] Li L., Zhuo H., Zhu J., Kwan A. (2019). Packing density of mortar containing polypropylene, carbon or basalt fibres under dry and wet conditions. Powder Technol..

[30] Nematollahi B., Vijay P., Sanjayan J., Nazari A., Xia M., Nerella V.N., Mechtcherine V. (2018). Effect of Polypropylene Fibre Addition on Properties of Geopolymers Made by 3D Printing for Digital Construction. Materials.

[31] Kumar R., Bhattacharjee B. (2003). Porosity, pore size distribution and in situ strength of concrete. Cem. Concr. Res..

[32] Das B., Kondraivendhan B. (2012). Implication of pore size distribution parameters on compressive strength, permeability and hydraulic diffusivity of concrete. Constr. Build. Mater..

[33] Cui L., Cahyadi J.H. (2001). Permeability and pore structure of OPC paste. Cem. Concr. Res..

[34] Zhou J., Ye G., van Breugel K. (2010). Characterization of pore structure in cement-based materials using pressurization–depressurization cycling mercury intrusion porosimetry (PDC-MIP). Cem. Concr. Res..

[35] Surendra P.S., Rangan B.V. (1971). Fiber Reinforced Concrete Properties. ACI J. Proc..

[36] Li V., Stang H., Krenchel H. (1993). Micromechanics of crack bridging in fibre-reinforced concrete. Mater. Struct..

[37] Li V. (2002). Large Volume, High-Performance Applications of Fibers in Civil Engineering. J. Appl. Polym. Sci..

[38] Kanavaris F., Azenha M., Soutsos M., Kovler K. (2019). Assessment of behaviour and cracking susceptibility of cementitious systems under restrained conditions through ring tests: A critical review. Cem. Concr. Compos..

